# Draft Genome Sequence and Biofilm Production of a Carbapenemase-Producing *Klebsiella pneumoniae* (KpR405) Sequence Type 405 Strain Isolated in Italy

**DOI:** 10.3390/antibiotics10050560

**Published:** 2021-05-11

**Authors:** Teresa Fasciana, Andrea Ciammaruconi, Bernardina Gentile, Paola Di Carlo, Roberta Virruso, Maria Rita Tricoli, Daniela Maria Palma, Giovanna Laura Pitarresi, Florigio Lista, Anna Giammanco

**Affiliations:** 1 Department of Health Promotion, Mother and Child Care, Internal Medicine and Medical Specialties, University of Palermo, 90127 Palermo, Italy; paola.dicarlo@unipa.it (P.D.C.); anna.giammanco@unipa.it (A.G.); 2 Scientific Department, Army Medical Center, 184 Rome, Italy; andrea.ciammaruconi@gmail.com (A.C.); dinagentile629@gmail.com (B.G.); romano.lista@gmail.com (F.L.); 3 Unita Operativa Complessa of Microbiology, Virology and Parassitology, A.O.U.P. “Paolo Giaccone”, 90127 Palermo, Italy; robivirruso@gmail.com (R.V.); mariaritatricoli@gmail.com (M.R.T.); laura.pitarresi968@gmail.com (G.L.P.); 4 II Intensive Care Unit, ARNAS “Civico, Di Cristina and Benfratelli”, 90127 Palermo, Italy; danipalma73@yahoo.it

**Keywords:** *Klebsiella pneumoniae*, carbapenemase, ST405, biofilm

## Abstract

Rapid identification and characterization of multidrug-resistant *Klebsiella pneumoniae* strains is essential to diagnose severe infections in patients. In clinical routine practice, *K. pneumoniae* is frequently identified and characterized for outbreak investigation. Pulsed-field gel electrophoresis or multilocus sequence typing could be used, but, unfortunately, these methods are time-consuming, laborious, expensive, and do not provide any information about the presence of resistance and virulence genes. In recent years, the decreasing cost of next-generation sequencing and its easy use have led to it being considered a useful method, not only for outbreak surveillance but also for rapid identification and evaluation, in a single step, of virulence factors and resistance genes. Carbapenem-resistant strains of *K. pneumoniae* have become endemic in Italy, and in these strains the ability to form biofilms, communities of bacteria fixed in an extracellular matrix, can defend the pathogen from the host immune response as well as from antibiotics, improving its persistence in epithelial tissues and on medical device surfaces.

## 1. Introduction

*Klebsiella pneumoniae*, bacteria of the family Enterobacteriaceae, is a main human pathogen causing hospital- and community-acquired infections such as bacteremia, urinary tract infections, pneumonia, and pyogenic liver abscesses [1,2].

Some *K. pneumoniae* isolates have been shown to be resistant to first-line antibiotics. Due to the spread of carbapenem-resistant *K. pneumoniae* (CR-*Kp*), this pathogen represents one of the microorganisms constituting an urgent threat to human health. Antimicrobial resistance to carbapenem is commonly related to the spread of transmissible plasmids, and the acquisition of resistance genes normally occurs by horizontal gene transfer [3]. Antimicrobial resistance plasmids have been called “epidemic resistance plasmids” because of their tendency to acquire resistance genes and the rapid diffusion among members of the family Enterobacteriaceae. Antimicrobial resistance determinants of epidemic plasmids offer a benefit to high-risk clones and are probably essential to their spread [4].

Resistance to carbapenems involves multiple mechanisms, including the following: alterations in outer membrane permeability mediated by the loss of porins, upregulation of efflux systems along with hyperproduction of AmpC β-lactamases, extended-spectrum β-lactamases (ESBLs), or more commonly, the production of carbapenemases.

Twenty-two different KPC (*Klebsiella pneumoniae* carbapenemase) enzyme variants have been identified. KPC β-lactamases can hydrolyze all β-lactams, including carbapenems, cephalosporins, cephamycins, monobactams, and clavulanic acid.

KPCs have been found in many Gram-negative species, including both Enterobacteriaceae and non-fermenters. KPCs are frequently found in *K. pneumoniae* associated with nosocomial infections, such as urinary tract infections, septicemia, pneumonia, and intra-abdominal infections.

The global spread of CR-*Kp* has been largely associated with “high-risk” carbapenemase-producing clones, mostly sequence type (ST) 258 and its related variants (clonal complex 258 (CC258)). However, in healthcare facilities, the spread of CR-*Kp* isolates other than ST258 has already been described [5,6,7]. In the geographic area of Palermo, Italy, CC258 is still prevalent; however, several other STs (e.g., ST307, ST395, ST392, ST348, ST405, and ST101) are emerging and circulating. This indicates a more complex polyclonal spread of *K. pneumoniae* carbapenemase producers [8,9,10].

For pathogen survival, other than the acquisition of resistance, virulent traits are also important, and some reports suggest that they may have an essential role in the pathogenesis of *K. pneumoniae* infections [11,12]. However, the most important virulence factors responsible for *K. pneumoniae* pathogenesis are capsular polysaccharides, which can collaborate to form biofilm [13,14], supporting the bacterial attachment to living or abiotic surfaces and preventing the effects of antimicrobial agents [15,16].

Using a next-generation sequencing (NGS) approach, it is possible to obtain molecular characterization in terms of genotyping and analyses of the genetic repertoire, including antibiotic resistance, virulence-related factors, and plasmid content of strains [17,18].

On the basis of this indication, the determination of genetic diversity for dominant species identification and complete knowledge of biofilm formation is necessary for stopping the spread of infection in hospitals and for the control and management of associated infections. In addition, knowing the principal genotype of isolates can aid in the identification of the cause of an infection in order to realize protective actions and infection control.

The purpose of this study was to report the draft sequence of a carbapenemase- and biofilm-producing *K. pneumoniae* strain belonging to ST405 isolated from a clinical sample.

We hypothesize that despite the in vitro resistance to the antibiotic therapy used, the infection could be resolved by the early use of combined therapy at high doses of antibiotics and by the immediate removal of the peripherally inserted central catheter (PICC).

## 2. Results

### 2.1. Molecular Genotyping

The in silico multilocus sequence typing (MLST) analysis revealed that the strain belonged to ST405. The image below shows the ST405 with respect to the carbapenem-resistant *K. pneumoniae* ST collected from March 2014 to March 2016 at the Azienda Ospedaliera Universitaria Policlinico “P. Giaccone” of Palermo [13].

**Figure 1:**
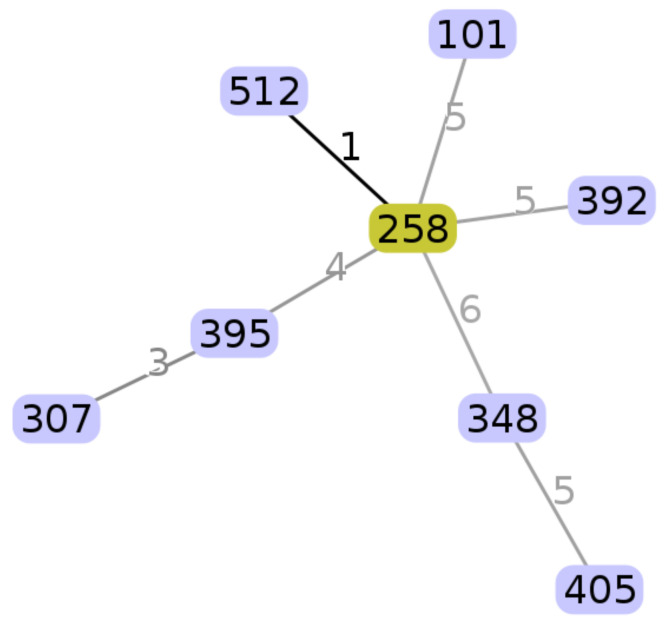
A radial diagram created using goeBURST.

In Figure 1, we report a radial diagram showing each sequence type (ST) represented by a circle with the numbers indicating the locus differences between the STs. The numbers in Table 1 show the corresponding housekeeping gene alleles for each ST.

A resistome study confirmed the susceptibility test results, and genes encoding for factors associated with resistance to the main classes of antibiotic were identified in silico. In particular, we found the presence of genes associated with resistance to beta-lactamase (*bla_KPC-3_*, *bla_CTX-M-15_*, *bla_OXA-1_*, *bla_SHV-76_*, *bla_TEM-1_*), aminoglycoside (*aac(3) lla-c-2*, *aac(6′)-Ib-cr*, *aph(3)pp Ib-2*, *aph(6) Id-1*), heavy metal (*pcoA-2*, *pcoB-3*, *pcoC-1*, *pcoD-2*, *pcoE-1*, *pcoR-1*, *pcoS-2*, *silC-3*, *silE-3*, *silR-2*, *silS-2*), quinolone (*gyrA-4*, *gyrB-1*, *parC-2*, *qnrB-1*), and efflux systems and regulators (*acrR-1*, *envR-15*, *fis-1*, *marA-2*, *marR-1*, *oqxR-4*, *rob-21*, *sdiA-6*, *soxR-2*, *soxS-4*).

The screening for (putative) virulence profile revealed the presence of a variety of virulence-associated genes. In particular, genes encoding for type-3 fimbriae, biofilm formation and host cell adherence (*mrkA-4*, *mrkB-1*, *mrkF-38*, *mrkH-15*, *mrkI-18*, *mrkJ-1*, and cps cluster genes, *wzi-143 and wzc-937*, associated with the K type (K) and the K locus (KL)-151), the *fyuA* yersiniabactin receptor, *irp1-148* and *irp2-145* aerobactin, *kvgA-2* regulator system component, yersiniabactin system (*ybtA-1*, *ybtA-39*, *ybtE-69*, *ybtP-4*, *ybtQ-60*, *ybtS-6*, *ybtT-39*, *ybtU-2*, *ybtX-62*), and microcin E492 system components (*mceA-1*, *mceC-1*, *mceD-3*, *mceE-2*, *mceH-5*) were detected.

The PlasmidFinder (http://www.genomicepidemiology.org) (accessed on 10 May 2021) database was used to describe the replicon plasmid content type of the *K. pneumoniae* isolate. Three plasmid Inc types were identified: IncFII(K) (CP000648), IncFIB(pQil) (JN233705), and IncFIB(K)-Kpn3 (JN233704). However, the intrinsic limits of short-read technologies (e.g., Illumina) in the WGS (Whole Genome Sequencing) method did not allow us to accurately reconstruct the genomic context surrounding the repeated sequences, typically related to antibiotic resistance and virulence determinants, in the plasmids.

The genes correlated with resistance and virulence are reported in Table 2.

### 2.2. Biofilm Formation Detection

In this study, the *K. pneumoniae* isolate tested was considered a moderate biofilm producer.

The obtained values of optical density (OD) of negative control, cut-off, ST 405, and two *K. pneumoniae* ATCC strains (ATCC 700603 and ATCC 13883 used as controls) are reported in Table 3.

## 3. Discussion

In summary, the retrospective WGS analysis of the *K. pneumoniae* isolate allowed us to define a comprehensive overview of the genetic profile, which is useful for gaining insights into the molecular characterization of antibiotic resistance, virulence potential, and plasmid content [19].

The identified virulence determinants may have contributed to the bacterial attachment/penetration and consequently to the infection and/or colonization severity of *K. pneumoniae*, which are very well adapted to their host environment.

The manifestation of adhesins is principally significant in the colonization phase, when mechanical forces such as peristalsis and salivary secretions impede bacterial attack of the host. The manifestation of type-3 fimbriae is known to be involved in biofilm development on biotic and abiotic sides of medical devices in a hospital environment. Capsules can also play a central role in *Klebsiella* spp. persistence inside and outside human hosts by repelling complement-mediated lysis or phagocytosis and contributing some defense against environmental dehydration. They may also have a counteracting effect on antibodies as a result of the release of excessive capsular material. Iron scavenging is significant in infections since hosts have little free iron under physiological settings, and the existence of multiple iron acquisition systems may confirm optimal iron acquisition in diverse host environments. Microcin E492, a bacteriocin active against members of the Enterobacteriaceae family, has been exposed to induce apoptosis in human cell lines, which may offer a benefit during gastrointestinal colonization by helping selective environmental fitness or playing a direct part in virulence [20,21].

Our results confirm that despite the in vitro resistance to the antibiotic therapy used, the patient survived the infection thanks to the early use of combined therapy at high doses of tigecycline and the immediate removal of the peripherally inserted central catheter (PICC). Retrospective studies that also included patients with severe infections due to *K. pneumoniae* (KPC-*Kp*) that produce *bla*_KPC_ have also described the importance of early onset of double and or triple combination therapy in reducing mortality at 14 days in pan-resistant isolates [22,23].

Finally, other studies have suggested that a double combination of CS (Colistin) plus TGC (Tigecycline) is synergic to reducing biofilms on in vitro catheter models, but only at high concentrations of both drugs is TGC effective in the reduction of biofilm cells [24,25].

## 4. Materials and Methods

### 4.1. Patient Information, Bacterial Isolation, and Identification

The strain was isolated from a peripherally inserted central catheter (PICC) applied in a 54-year-old female patient hospitalized in the University Hospital of Palermo, Italy, in 2014. The patient signed an informed consent form before the recovery.

The patient was admitted for emergency surgery on 16 September 2013, for colon surgery; after 18 days, she was transferred to the intensive care unit until 18 March 2014, when she was transferred to the internal medicine unit. After 5 days, a strain of *K. pneumoniae* was isolated from the PICC.

The same strain was isolated from all blood cultures performed.

At this time, *K. pneumoniae* sequence type (ST) 258 producing *K. pneumoniae* carbapenemase (KPC-*Kp*) bloodstream infection was reported in postoperative abdominal surgery patients [26]. Therefore, due to the patient’s critical condition and suspicion of surgical complication, an empiric double combination antibiotic treatment with high-dose tigecycline (100 mg every 12 h) and colistin at a dosage of 5 mg/kg/day divided in three equal doses was started, the PICC was removed, and a susceptibility test was requested. The patient was discharged on 13 May 2014.

A Becton-Dickinson Phoenix™ automated system (Becton Dickinson, Sparks, MD, USA) was used for species and antimicrobial susceptibility tests. Susceptibility profiles were understood according to the European Committee on Antimicrobial Susceptibility Testing breakpoints criteria, while susceptibility to colistin and tigecycline was confirmed by the MicroScan system (Beckman Coulter, Inc., Brea, CA, USA).

The strain showed resistance to multiple clinically used antibiotics: beta-lactams, aminoglycosides, antipseudomonal penicillin and beta-lactamase inhibitors, extended and non-extended spectrum cephalosporins, fluoroquinolones, sulfonamides, monobactams, penicillin and beta-lactamase inhibitors, antipseudomonal fluoroquinolones, and colistin. The isolate showed susceptibility for only tigecycline, as shown in Table 4.

### 4.2. Molecular Genotyping

The bacteria grown on blood agar for 18 h were used for the DNA extraction and for sequencing using the QIAmp^®^ DNA Mini kit Qiagen (QIAGEN GmbH, Hilden, Germany). A NanoDrop 8000 spectrophotometer (Thermo Fisher Scientific, Waltham, MA, USA) was used to analyze the quantity and purity of the DNA.

A Nextera XT Library Prep Kit (Illumina, San Diego, CA, USA) was used for library preparation and a NextSeq Mid-Output kit v2 (300-cycles) for sequencing on a NextSeq 500 platform (Illumina). The sequencing generated 3,534,792 paired-end reads checked for quality using FASTQC (https://www.bioinformatics.babraham.ac.uk/projects/fastqc/, accessed on 10 May 2021), trimmed with Sickle (https://github.com/najoshi/sickle, accessed on 10 May 2021), and assembled using SPAdes V3.7.0 [27]. The assembly yielded 207 contigs >500 bp with 137-fold coverage. The submitted sequences have a combined length of 5,571,183 bp with a G + C content of 58% and an N50 of 82,335 nucleotides. The contiguous sequences were annotated using the NCBI Prokaryotic Genomes Automatic Annotation Pipeline (PGAAP; http://www.ncbi.nim.nih.gov/genome/annotation_prok/, accessed on 10 May 2021).

The BIGSdb-Kp database (http://bigsdb.web.pasteur.fr/klebsiella/klebsiella.html, accessed on 10 May 2021) was used to conduct multilocus sequence typing (MLST) analysis and to define antibiotic resistance and virulence-related mechanisms.

Phyloviz software based on the goeBURST algorithm was used to visualize the evolutionary relationships among the isolates [28,29].

The PlasmidFinder-1.3 web tool (Center for Genomic Epidemiology, Lyngby, Denmark; http://www.genomicepidemiology.org (accessed on 10 May 2021); ID 95%) was used to define the replicon plasmid content type.

### 4.3. Quantitative Biofilm Production Assay

Three wells of a 96-well flat-bottomed plastic tissue culture plate were filled with 180 μL of Luria–Bertani (LB) supplemented with 1% glucose and 20 μL of overnight culture diluted to a final optical density of 600 (OD 600) = 0·1 were used for quantification of biofilm production. As a negative control, sterile LB supplemented with 1% glucose was used, while the strain type *K. pneumoniae* ATCC 13883 was selected as positive control. After incubation at 37 °C for 18 h, each well was washed three times with PBS, dehydrated for 1 h at 60 °C, and marked for 15 min with 180 μL of 2% Hucker’s crystal violet (Sigma-Aldrich Corporation, St. Louis, MO, USA). The dye bound to the adherent cells was solubilized with 180 μL of 33% (*v*/*v*) glacial acetic acid, and the absorbance was measured at 570 nm (OD 570). The assay was done in triplicate and repeated four times [30,31].

The OD cut-off (ODc) was defined as three standard deviations above the mean OD of the negative control.

The ability of biofilm formation was evaluated on the basis of the adherence capabilities into the following categories: non-biofilm producers (OD ≤ ODc), weak biofilm producers (ODc < OD ≤ 2 × ODc), moderate biofilm producers (2 × ODc < OD ≤ 4 × ODc), and strong biofilm producers (4 × ODc < OD).

## 5. Conclusions

In conclusion, the routine clinical implementation of real-time WGS and evaluation of the ability to produce biofilm in hospital settings would help to surveil nosocomial pathogens and alert clinicians to their presence. This would provide well-founded and punctual data on the emergence and spread of antibiotic resistance with the consequent possibility of preventing outbreaks by applying infection control procedures and implementing a targeted antimicrobial stewardship program. The enhancement of the surveillance program is even more essential in the case of strains with antibiotic resistance and virulence factor co-existence that could lead to life-threatening, untreatable, and invasive infections.

## Figures and Tables

**Table 1:** Housekeeping gene alleles for each sequence type (ST).

Locus	Putative Function of Gene	Lengh (bp)	ST 258	ST 512	ST 395	ST 101	ST 392	ST 348	ST 307	ST 405
*rpoB*	beta subunit of RNA polymerase B	501	1	1	1	1	4	15	1	4
*gap A*	Glyceraldehyde 3-phoshate dehydrogenase	450	3	54	3	2	3	2	4	2
*mdh*	Malate deydrogenase	477	1	1	2	1	6	20	2	62
*Pgi*	Phosphoglucose isomerase	432	1	1	4	5	1	10	52	3
*pho E*	Phosphoporine E	420	1	1	1	4	7	12	1	10
*inf B*	Translation initiation factor 2	318	3	3	1	6	4	1	1	1
*ton B*	Periplasmic energy trasducer	414	79	79	4	6	40	16	7	110

**Table 2:** Resistance genes and virulence factors detected by next-generation sequencing (NGS) analysis.

	Resistance Genes					
	Beta-Lactamase	Aminoglycoside		Heavy Metal	Quinolone	Efflux Systems and Regulator Systems
Gene alleles	*bla_KPC-3_*,*bla_CTX-M-15_*, *bla_OXA-1_*, *bla_SHV-76_*, *bla_TEM-1_*	*aac3 lla-c2*, *aac6p Ib-b-cr*, *aph3pp Ib-2*, *aph6 Id-1*	*pcoA-2*, *pcoB-3*, *pcoC-1*, *pcoD-2*, *pcoE-1*, *pcoR-1*, *pcoS-2*, *silC-3*, *silE-3*, *silR-2*, *silS-2*		*gyrA-4*, *gyrB-1*, *parC-2*, *qnrB-1*	*acrR-1*, *envR-15*, *fis-1*, *marA-2*, *marR-1*, *oqxR-4*, *rob-21*, *sdiA-6*, *soxR-2*, *soxS-4*
	**Virulence Factors**					
Gene alleles	type-3 fimbriae, biofilm formation, host cell adherence/capsule	yersiniabactin receptor	aerobactin/regulator system		yersiniabactin system	microcin E492 system components
Gene alleles	*mrkA-4*, *mrkB-1*, *mrkF-38*, *rkH-15*, *mrkI-18*,*mrkJ-1/wzi-143*, *wzc-937*	*fyuA*	*irp1-148*, *irp2-145/kvgA-2*		*ybtA-1*, *ybtA-39*, *ybtE-69*, *ybtP-4*, *ybtQ-60*, *ybtS-6*, *ybtT-39*, *ybtU-2*, *ybtX-62*	*mceA-1*, *mceC-1*, *mceD-3*, *mceE-2*, *mceH-5*

**Table 3:** Optical density (O.D.) values.

	OD 570
Negative control (median values)	0.059
Standard deviation	0.005
Cut-off (median values)	0.071
*K. pneumoniae* ST405 (median values)	0.215
ATCC 700603 (median values)	0.765
ATCC 13883 (median values)	0.102

**Table 4:** MIC values obtained.

Antibiotics	MIC Values µg/mL
IMP	>8
MEP	>8
ETP	>1
CIP	>1
AUG	>8/2
CXM	>8
CTX	>4
CAZ	>8
FEP	>8
SXT	>4/76
CN	>4
ATM	>16
TZP	>16/4
FOS	32
TGC	2
CS	4

MIC: minimal inhibitory concentration; IMI: imipenem; MEP: meropenem; ETP: ertapenem; CIP: ciprofloxacin; AUG: amoxicillin/clavulanic acid; CXM: cefuroxime; CTX: cefotaxime; CAZ: ceftazidime; FEP: cefepime; SXT: trimethoprim-sulfamethoxazole; CN: gentamycin; ATM: aztreonam; TZP: piperacillin/tazobactam; FOS: fosfomycin c/G6P; TGC: tigecycline; CS: colistin.

## Data Availability

The whole-genome shotgun project of *K. pneumoniae* has been deposited at GenBank, accession number SUB5047324, SAMN10765330.
